# Risk factors of stunting among children living in an urban slum of Bangladesh: findings of a prospective cohort study

**DOI:** 10.1186/s12889-018-5101-x

**Published:** 2018-01-30

**Authors:** M. Munirul Islam, Kazi Istiaque Sanin, Mustafa Mahfuz, A. M. Shamsir Ahmed, Dinesh Mondal, Rashidul Haque, Tahmeed Ahmed

**Affiliations:** 1 Nutrition and Clinical Services Division, International Centre for Diarrhoeal Disease Research, Bangladesh (icddr,b), Dhaka, 1212 Bangladesh; 2 Renal Unit, Menzies School of Health Research, Darwin, Australia; 3 Infectious Diseases Division, International Centre for Diarrhoeal Disease Research, Bangladesh (icddr,b), Dhaka, 1212 Bangladesh

**Keywords:** Stunting, Length at birth, Dietary diversity score, Urban slum, Bangladesh, Prospective cohort study, Generalized estimating equation

## Abstract

**Background:**

Bangladesh is one of the 20 countries with highest burden of stunting globally. A large portion (around 2.2 million) of the population dwells in the slum areas under severe vulnerable conditions. Children residing in the slums are disproportionately affected with higher burden of undernutrition particularly stunting. In this paper, findings of a prospective cohort study which is part of a larger multi-country study are presented.

**Methods:**

Two hundred and sixty five children were enrolled and followed since their birth till 24 months of age. Anthropometric measurements, dietary intake and morbidity information were collected monthly. Data from 9 to 12, 15–18 and 21–24 months were collated to analyze and report findings for 12, 18 and 24 months of age. Generalized estimating equation models were constructed to determine risk factors of stunting between 12 and 24 months of age.

**Result:**

Approximately, 18% of children were already stunted (LAZ < -2SD) at birth and the proportion increased to 48% at 24 months of age. Exclusive breastfeeding prevalence was only 9.4% following the WHO definition at 6 months. Dietary energy intake as well as intakes of carbohydrate, fat and protein were suboptimal for majority of the children. However, in regression analysis, LAZ at birth (AOR = 0.40, 95% CI: 0.26, 0.61), household with poor asset index (AOR = 2.81, 95% CI: 1.43, 5.52; ref.: average asset index), being male children (AOR = 1.75, 95% CI: 1.04, 2.95; ref.: female) and age (AOR = 2.34, 95% CI: 1.56, 3.52 at 24 months, AOR = 2.13, 95% CI: 1.55, 2.92 at 18 months; ref.: 12 months of age) were the significant predictors of stunting among this population.

**Conclusion:**

As the mechanism of stunting begins even before a child is born, strategies must be focused on life course approach and preventive measurement should be initiated during pregnancy. Alongside, government and policymakers have to develop sustainable strategies to improve various social and environmental factors those are closely interrelated with chronic undernutrition particularly concentrating on urban slum areas.

## Background

Stunting, which is defined as an attained height below − 2 standard deviations (SD) of the World Health Organization (WHO) growth reference median is the most prevailing form of child undernutrition and affects around 165 million children globally before their 5th birthday [1]. The deleterious effect of stunting is far-reaching beyond just shortened height and linked to future negative health and developmental consequences, such as impaired cognitive development, reduced economic productivity in adulthood, adverse maternal reproductive outcomes like obstructive labor and increased likelihood of development of non-communicable diseases [2]. The description of stunting as ‘chronic undernutrition’ may suggest a dietary origin of the problem, but the actual mechanism is extremely intricate including circumstances that lead to intrauterine growth restriction [3, 4], the household socio-economic condition and parental education [5–7], maternal and child undernutrition and recurrent infection [8] etc.

Although considerable growth faltering can occur during the prenatal period and the first 6 months of life, a significant proportion of stunting in low and middle income countries (LMICs) occurs within 6–24 months period [9–11] which is also known as complementary feeding period. Therefore this duration is considered to be crucial to mitigate stunting problem [12]. By definition, complementary feeding period is the time when children starts consuming a wide range of semi-solid to solid foods in addition to breast milk. Children < 2 years of age have greater nutrient needs to support rapid growth and cognitive development but have very limited gastric capacity compared to adults. To compensate this limitation, they must be fed complementary food high in nutrient density following recommended frequency by WHO. Unfortunately, the opposite is often practiced in LMICs [13] making these children susceptible to stunting.

Bangladesh has made exemplary improvement in achieving most of the millennium development goals (MDGs) over the last decade [14, 15]. Unfortunately, undernutrition among children < 5 years of age particularly stunting prevalence remains a challenging obstacle to overcome. According to UNICEF, Bangladesh is among the 20 countries in the world with the highest burden of stunting [16]. The latest national demographic and health survey reports that 36% children aged less than 5 years are stunted [17]. Furthermore, those living in urban slum areas are doing even worse as stunting prevalence is higher (50%) among them [18]. Dhaka the capital of Bangladesh, is one of the most densely populated cities in the world and almost 28% of this population lives in the slums [19]. With continuation of rapid urbanization, United Nations predicts that urban population of Bangladesh will grow by 50% during the next 14 years (2015–2029), and majority of people will live in urban areas [20]. Though this rapid urbanization is driven typically by a greater dynamic economic environment in the cities, for the same reason it attracts many of the poorest and disadvantaged population of rural society [3]. This disadvantaged population poses unique public health challenge in many ways including undernutrition. Yet there are very few published studies specifically focusing on chronic undernutrition and its predictors among children aged less than 2 years who are also slum dwellers. Therefore, the objective of our paper was to study the dietary practices of a cohort of children who were followed routinely since their birth till 24 months of age and to identify predictors of stunting among this population between 12 and 24 months of age using repeated longitudinal data in an urban slum context.

## Methods

### Study site and participant recruitment

Data for this paper was obtained from Bangladesh specific data of the study titled “The Interactions of Malnutrition & Enteric Infections: Consequences for Child Health and Development”, in short: the MAL-ED study. This is an ongoing multi-country study focusing on interaction between enteric infection and malnutrition [21]. The study site for Bangladesh is an urban slum area (Bawniabadh, Mirpur) in Dhaka, the capital of the country. This site was selected for its typical urban slum setting with poor socioeconomic and environmental context. The MAL-ED study in Bangladesh includes three components: (1) birth cohort component, (2) case-control component, and (3) twin studies. Data from birth cohort component was used to analyze and report in this paper. A preliminary census was performed to identify pregnant women in the community of Bawniabadh, Mirpur slum area in 2009. Participants were identified following predetermined inclusion and exclusion criteria. The inclusion criteria were caregiver had no plans to move out of the catchment area for at least 6 months following enrollment and willingness of caregiver to be visited in the home monthly. Exclusion criteria for cohort recruitment were maternal age < 16 years, not a singleton pregnancy, another child already enrolled in the MAL-ED study, severe illness of the child (who is supposed to be enrolled) requiring hospitalization prior recruitment, and severe acute or chronic conditions of the child diagnosed by a physician (e.g., neonatal disease, renal disease, chronic heart failure, liver disease, cystic fibrosis, congenital conditions). All children delivered in the study community meeting all the inclusion and none of the exclusion criteria were eligible and invited to enroll in the study between February 2010 and February 2012. The MAL-ED study field-workers visited the cohort households just after delivery to screen mothers and children for possible entry into the study. A study physician examined the child before enrollment for any apparent clinical/subclinical signs of illness and certified the child as healthy. Newborns were recruited with equal ratio of male and female. At enrollment, each child’s date of birth and sex were recorded; information about initiation of breastfeeding was collected; and the child’s length, weight, and head circumference were measured by two trained study staffs. Dietary Information of these children was collected monthly from 9 months onwards up to 24 months of age. Anthropometric and morbidity information were also collected on regular monthly basis. Information regarding morbidity covered the entire month since the last visit.

### Anthropometric measurement

The first anthropometric measurement was performed within 72 h after birth. Monthly anthropometric measurements were conducted by two trained field staffs following standard procedures. Children were weighed with minimum clothing using a digital scale with 10 g precision (Seca, model no.345, Hamburg, Germany) and recumbent length (children <2y) was measured using measuring board (Seca Infantometer, model no.417, Hamburg, Germany) to the nearest 1 mm according to standard anthropometric methodology [22]. The new WHO growth standards (2006) [23] was used to calculate different anthropometric indices: weight-for-age, z-score (WAZ), length-for-age, z-score (LAZ) and weight-for-length, z-score (WLZ). Based on these standards, stunting was defined as LAZ less than two standard deviations below the age specific medians [24]. To maintain quality of measurement, weighing and length measuring equipment were calibrated daily with standard weights and measured rods, and refresher training was provided periodically.

### Procedure of dietary assessment

We applied a multiple pass 24 hour (24-h) recall approach to collect dietary intake data monthly starting from 9 months of age of the study participants and onwards. This is an ideal technique to quantify energy and nutrient intakes of young children over time [25]. As a single 24-h recall does not provide a precise estimate of usual intake due to within-subject (i.e. Day to day variation) [26, 27], we have combined the data of dietary intakes over 9–12, 15–18 and 21–24 month time period to represent the usual intake as described in the original design of the study [28]. Averaging dietary data from four 24-h recall has enabled us to estimate the usual dietary macronutrient intake as dietary data collected on several days (3 or more recalls) is superior to construct usual nutrient intake [29]. Research staff trained by experienced dietitians conducted the 24-h recall interview [30] with the use of visual aids (standardized household measuring utensils and food pictures of various portion sizes) to assist the mothers to quantify their child’s dietary intake. The interviews took place on non-consecutive days and the participants were not notified in advance to evade any biasness. A locally adapted food composition table (Table 1) was used to convert the dietary intake data into nutrient data. To ensure maximum quality of the data collection technique, a secondary dietary recall with 10–20 randomly selected participants was conducted monthly.

**Table 1:** Sources of food composition table

1. USDA20-US Department of Agriculture Standard Reference Version 20 (2007)
2. World Food Dietary Assessment System, UC Berkeley
3. Food and Nutrient Database for Dietary Studies, Version 3.0 (2008)
4. NDS-Nutrient Data System for Research
5. Manufacturer label
6. Recipes based on information collected from homes or from local recipe books

### Operational definitions

#### Exclusive breastfeeding (EBF)

EBF has been defined according to WHO guideline [31] which states no other food or drink, not even water, except breast milk (including milk expressed or from a wet nurse) for first 6 months of life, but allows the infant to receive oral rehydration solution (ORS), drops and syrups (vitamins, minerals and medicines) as and when necessary.

#### Dietary diversity score and minimum dietary diversity

We estimated dietary diversity score (DDS) and minimum dietary diversity (MDD) based on seven food groups described in Bangladesh demographic and health survey (BDHS) 2014 [17]. The food groups are 1) starchy staples (grains, roots, and tubers); 2) legumes and nuts; 3) dairy products (milk yogurt, cheese); 4) flesh foods (meat, fish, poultry, and liver/organ meat); 5) eggs; 6) vitamin A-rich fruits and vegetables; and 7) other fruits and vegetables. If the children consumed any amount of food from these food groups on the previous day, it was scored as “1” (i.e., there was no minimum quantity). Food items not included in the mentioned groups were excluded during scoring. DDS for each child was calculated by summing the number of food groups consumed during 24-h recall period. We defined MDD as consuming foods from at least 4 different food groups (out of 7 food groups) over past 24 h. This cutoff was selected because it is estimated to be associated with better quality diets for both breastfed and non-breastfed children [32, 33]. At the same time, consumption of foods from at least 4 food groups has a high likelihood of consuming at least one animal source food and at least one fruit or vegetable in addition to a staple food (grains, roots, or tubers) [34].

### Variable selection for analysis

Different tiers of determinants of stunting such as inherent, proximal, intermediate and distal have been reported in previously published studies [35, 36]. We selected the variables based on such determinants and availability of data from our study (Table 2). Considering repeated longitudinal manner of data, the outcome variable was defined as stunting at 12, 18 and 24 months of age. Nutrient intake data has been averaged over 9–12, 15–18 and 21–24 months of age and analyzed from four 24-h recalls. Number of days a child suffered from diarrhoea over 9–12, 15–18 and 21–24 months have been summed and presented as average number of days suffered from diarrhoea per month. Maternal education has been categorized into 3 groups as no schooling, minimum 5 years and more than 5 years of schooling. Toilet with flush facility and pit latrine with slab have been coded as improved toilet. The household asset index has been constructed using household asset data obtained from the socio-economic status questionnaires. Principal components analysis was performed to produce a common factor score for each household and depending on the score, they were further categorized into low (poor), intermediate, and high (wealthy) households.

**Table 2:** Selection of predictors and outcome variables

Factors	Explanatory variables	Outcome
Inherent	Age, sex	Stunting between 12 and 24 months of age
Proximal	Total days of exclusive breastfeeding during the first six months of life, average number of days suffered from diarrhoea/month, average dietary diversity score at 12, 18 and 24 months of age, proportion of calorie coming from complementary foods (carbohydrate and protein) at 12, 18 and 24 months of age	
Intermediate	Maternal age, Length for age Z-score at birth, Weight for	
	age Z-score at birth, improved toilet, drinking water source	
Distal	Maternal education, household asset index	

### Ethical consideration

The study has been approved by the Research Review Committee and Ethical Review Committee of the International Center for Diarrhoeal Disease Research, Bangladesh (icddr,b). Mothers/respective caregivers of participating children kindly provided the written consent. They were informed explicitly regarding the purpose of the study and were also informed that participation in such study was voluntary and had complete rights for non-response to the questionnaire or study related activities. They were given full rights to withdraw their children participating from the study at any time. Furthermore, at each follow-up visit, mothers/primary caregivers provided verbal consent on behalf of the children. All information and related records were anonymously entered in computer and analyzed without indicating the child’s name and identity. All the investigators obtained the certificate for “Human Participants Protection Education for Research Teams” through online course, sponsored by the National Institutes of Health.

### Data analysis

Data were entered and analyzed using SPSS for Windows (IBM SPSS Statistics V22.0). Anthropometric data were analyzed using WHO Anthro software (Geneva, Switzerland). For normally distributed continuous variables mean and standard deviation and for non-normally distributed continuous variables median and interquartile range (IQR) were reported. For multivariable regression, multicollinearity between independent variables were explored from correlation matrix and variance inflation factor (VIF) values using the collinearity diagnostics from linear regression command as collinearity statistics in regression focus on the relationships among predictors only [37]. Predictor variables with VIF value greater than 5 were screened again for correlation and number of similar variables were reduced in the subsequent models to lower the VIF value less than 1.5 to avoid collinearity.

To investigate association between outcome and predictor variables, we have used generalized estimating equation (GEE) models to account for correlation between outcomes at different follow-up times from the same child. We used generalized estimating equation options from generalized liner models menu of SPSS and generated odds ratios and corresponding 95% confidence interval (CI), using data from 9 to 12, 15–18 and 21–24 months of age. We constructed the GEE models using both autoregressive (AR1) and unstructured covariance matrix with robust variance estimates. Model goodness of fit was determined based on lowest quasi likelihood under independence model criterion (QIC) value. The unstructured covariance matrix provided slightly higher QIC value compared to AR1 covariance matrix (778.5 VS 775.9 respectively) but lower standard error values for the coefficients providing better precision. Therefore we used the unstructured covariance matrix to construct the final models. Categorical predictor variables (sex, age, maternal education, presence of improved toilet, water source and household asset) were selected as factors and continuous variables (LAZ at birth, WAZ at birth, total days of exclusive breastfeeding during the first six months of life, average number of days the child suffered from diarrhoea, dietary diversity score, maternal age, proportion of calorie coming from carbohydrate and protein portion of complementary food) were treated as co-variates. Proportion of calorie coming from fat portion of the complementary food was not included in the model as a co-variate due to high multicollinearity with the variable expressing proportion of calorie coming from carbohydrate portion. Initially each predictor variable was used individually in the GEE model to identify its unadjusted effect on the outcome variable (stunting). In the subsequent models, all the predictor variables were entered concurrently to obtain adjusted final model. A probability of less than 0.05 was considered statistically significant during analysis. The strength of association was determined by estimating the adjusted odds ratios (AOR) and their 95% CIs.

## Result

**Fig. 1:**
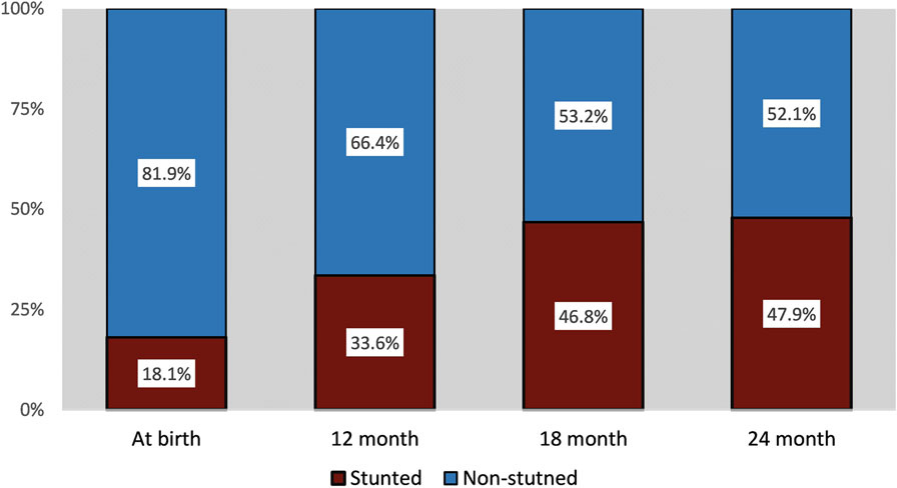
Proportion of stunting from birth till 24 months of age. Stunting defined as length for age Z score < -2SD

A total of 265 children were enrolled and followed up in this cohort study since birth till 24 months of age. Average age of study participant’s mothers was around 25 years (SD 4.9) and 18.6% mothers had no schooling whereas 44% had schooling for at least 5 years. Approximately 35% households were poor but 76% of all households used improved toilet. Among the study participants, 51.3% were female. Average birth weight was 2.75 kg (SD 0.41) and average length was 48.05 cm (SD 2.05). Their mean LAZ and WAZ scores at birth were − 1.08 (SD 1.02) and − 1.31 (SD 0.92) respectively. At birth approximately 18% children were stunted (LAZ < -2SD). This proportion increased progressively over time and by the age of 24 months, almost 48% of the participants were stunted (Fig. 1) with overall mean LAZ of − 2.03 (SD 0.93).

Only 9.4% children were exclusively breastfed for complete 6 months (180 days) with mean 98.65 days (SD 57.40) of EBF for the whole cohort. Among all the children, 45.3% were exclusively breastfed for complete 3 months and another 45.3% had exclusive breastfeeding for more than 3 months but less than 6 months. At the age of 24 months, approximately 89% of the children were still breastfed. Average breastfeeding frequency was approximately 12 times/day at 12 months of age which reduced to 10 times/day at 18 months and 8 times/day at 24 months of age. On an average children suffered from diarrhoea for 5.62 (SD 5.92), 4.03 (SD 4.94) and 3.14 (SD 5.61) days per month at 12, 18 and 24 months of age respectively. Characteristics of the children according to their stunting status are given in Table 3.

**Table 3:** Characteristics of study participants according to stunting status at respective age

	At birth		At 12 month		At 18 month		At 24 month	
	*n* = 265		*n* = 229		*n* = 218		*n* = 211	
	Stunted	Non-stunted	Stunted	Non-stunted	Stunted	Non-stunted	Stunted	Non-stunted
	48	217	77	152	102	116	101	110
Sex	(F = 27, M = 21)	(F = 109, M = 108)	(F = 35, M = 42)	(F = 79, M = 73)	(F = 49, M = 53)	(F = 53, M = 63)	(F = 44, M = 57)	(F = 59, M = 51)
Mean LAZ	−2.64 ± 0.56	−0.74 ± 0.74	−2.67 ± 0.59	−1.14 ± 0.58	−2.74 ± 0.59	−1.25 ± 0.53	− 2.80 ± 0.60	− 1.32 ± 0.53
Mean WAZ	− 2.34 ± 0.72	− 1.08 ± 0.80	−1.99 ± 0.77	−0.81 ± 0.91	− 2.14 ± 0.70	−0.92 ± 0.89	−2.22 ± 0.73	−1.05 ± 0.85
ABF			11.31 ± 3.73	12.28 ± 2.82	10.19 ± 3.99	10.33 ± 3.07	7.70 ± 4.32	8.55 ± 3.58
ADD			6.45 ± 6.21	5.43 ± 5.80	5.01 ± 5.691	3.34 ± 4.03	3.84 ± 7.14	2.67 ± 3.80

*LAZ* Length for age Z score

*WAZ* Weight for age Z score

*ABF* Average breastfeeding frequency/day

*ADD* Average days suffered from diarrhea/month

**Fig. 2:**
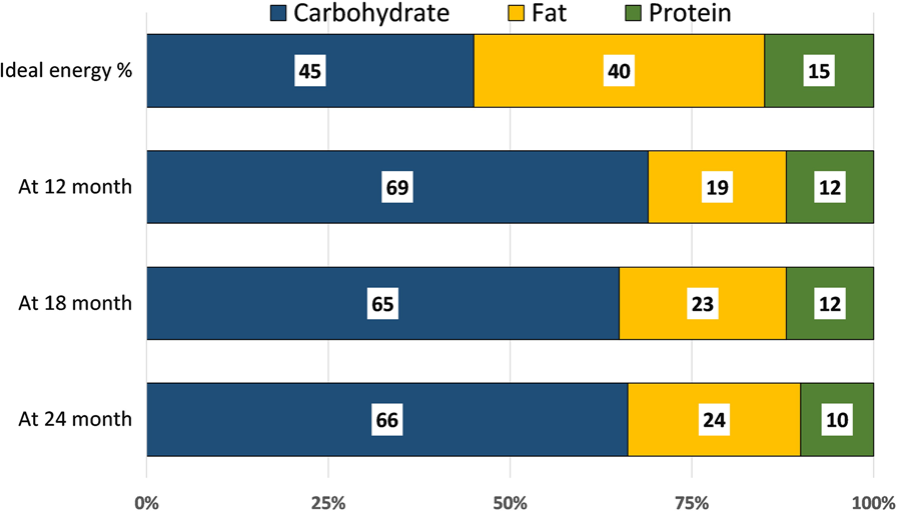
Proportion of energy coming from macronutrients at different ages from complementary foods

The median energy consumption from complementary food among the study children was 181 kcal/day (IQR 125, 256) at 12 months of age that subsequently increased to 318 kcal/day (IQR 228, 401) at 18 months and 484 kcal/day (IQR 380, 617) further at 24 months. Average protein consumption was 5.34 g/d (IQR 3.38, 7.87), 9.19 g/d (IQR 6.35, 11.73) and 13.42 g/d (IQR 10.29, 16.28) at 12, 18 and 24 months of age respectively (Table 4). However, of these consumptions, the average animal-protein consumptions were only 1.89 g/d, 3.51 g/d and 4.68 g/d at 12, 18 and 24 months respectively. At 12 months, the mean DDS was 3.51 (SD 0.91), which increased to 4.09 (SD 0.86) at 18 months and 4.53 (SD 0.81) at 24 months of age. Approximately 33% of the children at 12 months of age consumed at least 4 or more food groups from selected 7 food groups (MDD). Proportion of children with MDD increased subsequently over period of time and approximately 60% children at 18 months and 79% children at 24 months of age consumed 4 or more food groups out of 7 food groups. The average consumption of different macronutrients (protein, carbohydrate, fat) from complementary foods according to stunting status at respective ages is provided in Table 4. Overall, at 12 months, 69% of the dietary energy came from carbohydrate, 19% from fat and 12% from protein (Fig. 2). Similarly at 24 months of age, dietary carbohydrate, fat and protein were source of 66%, 24% and 10% of total energy consumption from complementary food respectively.

**Table 4:** Average macronutrient intake from complementary foods at different ages according to stunting status

	RDA	At 12 months			At 18 months			At 24 months		
		All	Stunted	Non-stunted	All	Stunted	Non-stunted	All	Stunted	Non-stunted
		(*n* = 229)	*n* = 77)	*n* = 152)	(*n* = 218)	(*n* = 102)	(*n* = 116)	(*n* = 211)	(*n* = 101)	(*n* = 110)
Energy		181.32	177.02	189.52	317.97	310.95	322.66	483.57	466.20	502.55
(kcal/d)		(124.99, 255.84)	(126.93, 232.92)	(123.71, 302.47)	(227.51, 401.31)	(233.32, 404.63)	(221.47, 387.30)	(380.05, 616.55)	(368.49, 621.51)	(395.48, 616.66)
Protein	13	5.34	5.18	5.85	9.19	8.84	9.29	13.42	12.92	13.48
(g/d)		(3.38, 7.87)	(3.58, 7.76)	(3.25, 8.45)	(6.35, 11.73)	(6.19, 11.53)	(6.65, 12.15)	(10.29, 16.28)	(10.23, 16.86)	(10.32, 15.99)
Carbohydrates	130	31.09	29.77	32.75	51.06	50.12	51.29	79.35	73.12	82.3
(g/d)		(21.72, 42.71)	(22.22, 40.42)	(21.55, 52.20)	(38.23, 66.26)	(39.98, 66.06)	(37.65, 68.15)	(62.20, 100.22)	(61.59, 99.79)	(63.54, 101.47)
Fat	ND	3.89	3.69	3.90	7.79	7.55	8.09	13.13	13.10	13.12
(g/d)		(2.50, 6.21)	(2.48, 6.94)	(2.53, 6.15)	(5.19, 11.13)	(5.20, 11.27)	(5.25, 11.14)	(8.93, 17.45)	(8.40, 17.49)	(9.2, 17.41)
Animal protein^a^		1.89	1.69	2.01	3.51	3.14	3.9	4.68	4.55	4.79
(g/d)		(0.86, 3.88)	(0.57, 3.86)	(1.07, 3.83)	(1.81, 5.88)	(1.41, 5.67)	(2.07, 6.10)	(2.91, 7.34)	(2.96, 7.50)	(2.87, 6.73)
Phytate		49.16	55.27	48.41	90.61	92.49	85.73	164.10	172.51	150.19
(mg/d)		(33.36, 73.53)	(31.20, 78.42)	(33.39, 70.03)	(64.68, 132.38)	(68.07, 141.27)	(65.09, 128.95)	(121.45, 218.53)	(131.63, 225.57)	(118.53, 206.25)
DDS		3.51 ± 0.91	3.43 ± 1.06	3.56 ± 0.84	4.09 ± 0.86	3.86 ± 0.83	4.29 ± 0.80	4.53 ± 0.81	4.44 ± 0.81	4.63 ± 0.80

*RDA* Recommended dietary allowance (An RDA is the average daily dietary intake level; sufficient to meet the nutrient requirements of nearly 97–98% healthy individuals in a group)

*ND* Not determined. *DDS* Dietary diversity score (Mean ± SD)

^a^Values are median and interquartile ranges unless otherwise stated

Results from the unadjusted and adjusted GEE models to identify factors associated with stunting are given in Table 5. WAZ score at birth, number of days of exclusive breastfeeding during the first six months of life, average days suffered from diarrhoea per month, proportion of calorie coming from either carbohydrate or protein portion of complementary food and dietary diversity score, none of these variables were predictive of stunting between age of 12–24 months but age, sex, LAZ score at birth and household asset index were. Being male had almost 2 times higher odds of being stunted compared to being female (AOR = 1.75, 95% CI: 1.04, 2.95). One unit increase in LAZ score at birth had 60% lower likelihood (AOR = 0.40, 95% CI: 0.26, 0.61) of being stunted between 12 and 24 months of age. Furthermore, compared to households with intermediate or average asset index, children from poor households had almost 3 times higher odds of being stunted (AOR = 2.81, 95% CI: 1.43, 5.52). Age also had significant effect on stunting as there was 2 times higher likelihood of being stunted at 18 months and 24 months of age compared to 12 months, (AOR = 2.13, 95% CI: 1.55, 2.92) and (AOR = 2.34, 95%CI: 1.56, 3.52) respectively. We also assessed the effect of several variables like; average macronutrient (carbohydrate, protein, fat) intakes in grams per day,, exclusively breastfed for 6 months (“yes” or “no”), average number of breastfeeding frequency per day, status of minimum dietary diversity (received minimum diversified food = “yes” or “no”) individually and simultaneously using multiple GEE models on stunting as outcome. We also have explored interaction between age and average intake, gender and household asset index in separate models. However, none of these models were statistically significant and did not construct good predictive models and therefore not presented here.

**Table 5:** Factors associated with stunting at 12–24 months of age- results from unadjusted and adjusted GEE models^a^

Predictors	Unadjusted OR (95% CI)	*p* value	Adjusted OR (95% CI)	*p* value
Inherent factors				
Age				
24 months	1.76 (1.38, 2.25)	0.000	**2.34 (1.56, 3.52)**	**0.000**
18 months	1.71 (1.36, 2.16)	0.000	**2.13 (1.55, 2.92)**	**0.000**
12 months	Ref			
Sex				
Male	1.32 (0.83, 2.10)	0.244	**1.75 (1.04, 2.95)**	**0.036**
Female	Ref			
Proximal factors				
DEBF	1.002 (0.99, 1.01)	0.339	1.00 (0.99, 1.01)	0.946
ADD	1.00 (0.98, 1.02)	0.844	1.01 (0.99, 1.04)	0.339
DDS	1.09 (0.94, 1.26)	0.248	0.93 (0.74,1.16)	0.505
Proportion of calorie from CHO	0.99 (0.98, 1.01)	0.433	0.99 (0.97, 1.02)	0.737
Proportion of calorie from Protein	0.99 (0.94, 1.05)	0.852	0.99 (0.91, 1.09)	0.948
Intermediate factors				
Maternal age	1.00 (0.96, 1.05)	0.861	0.99 (0.94, 1.05)	0.802
LAZ at birth	0.41 (0.31, 0.54)	0.000	**0.40 (0.26, 0.61)**	**0.000**
WAZ at birth	0.51 (0.39, 0.66)	0.000	1.05 (0.68, 1.63)	0.814
Presence of improved toilet				
Yes	0.99 (0.54, 1.82)	0.977	0.83 (0.38, 1.82)	0.642
No	Ref			
Water source				
Piped to dwelling	2.18 (1.15, 4.12)	0.017	1.42 (0.73, 2.77)	0.304
Piped to plot	Ref			
Distal factors				
Maternal education				
> 5 years	0.64 (0.32, 1.27)	0.202	0.72 (0.31, 1.65)	0.432
≤ 5 years	0.79 (0.41, 1.52)	0.479	0.84 (0.40, 1.75)	0.642
No schooling	Ref			
Household asset				
Wealthy	1.55 (0.78, 3.08)	0.213	1.84 (0.93, 3.65)	0.080
Poor	2.95 (1.49, 5.82)	0.002	**2.81 (1.43, 5.52)**	**0.003**
Intermediate	Ref			

*OR* Odds ratio

*CI* Confidence interval

*DEBF* Total days of exclusive breastfeeding during the first six months of life

*ADD* Average number of days suffered from diarrhoea/month

*DDS* Dietary diversity score

*LAZ at birth* Length for age Z-score at birth

*WAZ at birth* Weight for age Z-score at birth

^a^GEE models were constructed using unstructured correlation matrix with robust estimator. Included repeated observation from 9 to 12, 15–18 and 21–24 months of age and reported as average at 12, 18 and 24 months of age. Outcome variable- Stunting (LAZ < -2SD) as binary outcome between 12 and 24 months of age. Predictor variables are selected according to inherent, proximal, intermediate and distal factors associated with stunting, significant predictors in adjusted model are highlighted in bold

## Discussion

Around 2.2 million people in Bangladesh are currently living in 14,000 slums [38] and children residing in these areas remain the most underprivileged [39]. Most of the studies on dietary intake were carried out in rural settings of Bangladesh with cross-sectional design and unfortunately, there is a dearth of evidence regarding the risk factors for undernutrition particularly stunting in urban slum context. We observed a cohort of children living in an urban slum area to evaluate the average macronutrient intake of the children living in vulnerable environment and to ascertain risk factors associated with stunting between 12 and 24 months of age among this group. We found that being a male child, age, lower LAZ score at birth and being a member of poor household (at baseline) were associated with higher odds of being stunted between 12 and 24 months of age. However in this cohort of children, proportion of intake of calorie either from carbohydrate or protein portion of complementary food, dietary diversity score and duration of exclusive breastfeeding during first six months of life were not predictors of stunting.

Though Bangladesh has made remarkable progress in health sector, undernutrition is still an immense challenge for the country. The scenario is even worse in urban slum areas, as prevalence of stunting is higher in this vulnerable population. Overall the level of stunting among children under 5 years has decreased from 41% in 2011 to 36% in 2014 [17]. Nonetheless, half of the under-five children in slums are stunted (height-for age Z score < -2SD), compared to one-third in non-slum areas [18]. Similar to our study, national micronutrient survey 2013 reported higher prevalence (51.1%) of stunting in the urban slum areas of Bangladesh [40].

The mechanism of poor anthropometric outcome, particularly stunting is extremely intricate ranging from biological and social to environmental factors. A number of proximal (EBF, complementary feeding, newborn health status), intermediate (environmental status, maternal/reproductive health) and distal factors (maternal education, household asset) have been conceptualized to play interrelating role resulting in stunting [41]. It also has been established that the mechanism that causes stunting often starts very early in life, typically in utero, and continues during the first two post-natal years. Analogous to these evidences, in our prospective cohort study we have found that LAZ at birth, household asset, age and sex were significant predictors of stunting between 12 and 24 months of age even after controlling for other predictors.

Measurement of length at birth has been proved to be problematic due to technical and procedural issues. However, there is an absolute requirement for early measurement of length in certain circumstances, such as in the construction of growth curves [42]. Several studies have measured this indicator successfully as this is often part of protocols in long-term cohort studies [43–46] or short-term, longitudinal observational studies [47–53] trying to identify association between early growth and several health outcomes. A large cohort study with 3267 children done in rural Bangladesh [48, 54] found that average birth length of the children were 47.8 cm (SD 2.1), which is comparable to our study (48.05 cm, SD 2.05). A study from Indonesia where stunting prevalence was high (24% at 12 months of age), reported average birth length of the studied children as 49.7 cm (SD 2.2) [52] similar to this study.

We further found significant association between LAZ at birth and stunting at later ages after controlling for other factors as one standard deviation increase in LAZ had 60% reduced the likelihood of being stunted between 12 and 24 months of age (Table 5). We also found association between WAZ at birth with stunting but significance of the association disappeared after controlling for LAZ at birth suggesting that LAZ at birth is strong predictor of stunting. Similar findings have been suggested by other studies from countries with high prevalence of stunting. The Indonesian study concluded that neonatal length was the strongest predictor of infant nutritional status [52]. Another Peruvian birth cohort study reported that, each standard deviation decrease in LAZ at birth was associated with a 9.7 times greater odds of stunting among adolescents [46]. Berngard et al. reported that birth length was the major predictor of early infant linear growth failure in a longitudinal study done in Western Highlands of Guatemala. [55]. Another Guatemalan cross-sectional study concluded that stunting at birth must be recognized as a substantial driver of linear growth faltering in such context where stunting prevalence is high as the mechanism begins in utero [56]. Furthermore, Corvalán et al. concluded from their prospective study that higher length at birth was associated with increased fat free mass in adult [57]. Measurement of birth length has been reviewed skeptically and birth weight rather than birth length has become variable of interest in clinical medicine and public health. In the epidemiological studies, intrauterine growth retardation based on birth weight is the paradigm of public health interest [58]. This is evident from findings of a recent comprehensive study that performed global comparative risk assessment using country-level data from 137 developing countries which concluded that fetal growth restriction (defined as being term and small for gestational age) is the leading risk factor for stunting globally, followed by unhygienic sanitation [59]. In our study we have found both birth weight and length in their standardized form as significant predictor of stunting individually, but while all other predictor variables were controlled, standardized birth length remained significant rather than birth weight suggesting that if measured properly length at birth could identify children at risk of stunting.

We found that children from poor households were at significant higher risk of being stunted between 12 and 24 months of age compared to households with average asset index suggesting that the disparity among poor households is most severe. Socio-economic status based on household assets represent the long-run accumulation of household wealth [60], and several studies have reported strong association of asset index with child health outcomes [61, 62]. Poorest households are more likely to suffer from food and nutritional insecurity due to lack of resources, low levels of education and nutritional health information, and poor access to and utilization of healthcare [63, 64]. Poverty plays a big role in affecting child malnutrition and similar to many health indicators, children’s nutritional status is expected to be poor among urban slum population since poverty, coupled with environmental hazards, is likely to cause synergistic hazardous effects on children.

We did not find any significant association between presence of improved toilet or water source and stunting at 12–24 months of age, which is contrary to some reported observational studies [65, 66]. Such difference may be explained by difference in sample size and studied population. Studies by Rah and colleagues [65] and Fink and colleagues [66] have used large survey data consisting of both urban and rural population, whereas our study population came from a confined slum area where access to improved toilet and water is not very diverse. Nonetheless, association between stunting and these socio-economic factors has great implication particularly in view of urban slum context of Bangladesh since, more than 2 million people are living in different slums of the country [38] and the number is growing rapidly. It has been reported that, slum children in Bangladesh are most disadvantaged and worst performing in terms of children’s health and nutrition compared to rural and non-slum urban areas [39]. Therefore, policymakers need to lend their dedicated attention specifically to this vulnerable and growing population in order to tackle stunting issue in the country.

Combination of optimal breastfeeding and complementary feeding is critical to improve child survival and promote healthy growth and development. In the developing countries where stunting is exceedingly prevalent, promotion of breastfeeding and appropriate complementary feeding could prevent about 220,000 deaths among children under 5 years of age [67]. The total energy requirements of healthy, breastfed infants are approximately 686 kcal/d at 9–11 months, and 894 kcal/d at 12–23 months of age [68]. Among breastfed children in developing countries, average breast milk energy intake is 379 and 346 kcal/d at 9–11 and 12–23 months, respectively [69]. Therefore, the rest of the required energy for infants with “average” breast milk intake should be contributed by complementary foods [69]. Kimmons et al. [70] in their study in rural Bangladesh found that mean calorie intake from complementary food was 147 kcal/day (SD 102) at 12 months of age and concluded that total energy intake was lower than desirable. At the same age, our study participants consumed on an average 181 kcal/day (IQR 125, 256) which is close to the previous study and at 24 months of age dietary calorie consumption was 484 kcal/day (IQR 380, 617). Quantitative studies in low-income countries [71–73] have found that older infants and young children often consume substantially less than the recommended amount of energy from food [74]. Infants with increasing age require energy dense food to meet their physiological need for proper growth and development. Due to limited gastric capacity, it is essential that they must consume high energy dense food comparing to adults which is often simply opposite in the context of the developing countries [13] like Bangladesh.

According to our finding, 60% children in our cohort study consumed at least 4 or more food groups out of 7 food groups at 18 months of age and this proportion increased to 79% at the age of 24 months. Yet average calorie intake, protein specifically animal source protein and fat consumption were sub-optimal between 12 and 24 months of age. This clearly suggests poor quality of complementary feeding practices among our study participants which is also a common practice in rural areas [70, 75, 76] and in urban slums [77] or nationally as a whole [78, 79]. We also measured the dietary diversity score of the study participants but it was not a predictive factor of stunting in our GEE model. However, other published studies differ from our finding because many of them found low dietary diversity as a significant predictor of stunting [75, 76, 80]. The difference in findings between our study and others could be a result of different study designs as these studies applied cross-sectional design or survey whereas ours was a longitudinal prospective study and conducted in different settings. Also participants from other Bangladeshi studies came from rural population which is different from urban slum population as in our study. Food consumption patterns are affected by socio-cultural factors, determining food availability, access and utilization. Furthermore, such dietary score tends to be misleading in urban slum context where diets may appear diverse due to easy access to convenience food, but quantities of meat or dairy products consumed might be negligible [81].

In our study, stunting prevalence increased over time as well as male children were more likely to become stunted. According to latest BDHS, prevalence of stunting increased with age, as 14% children less than 6 months of age were stunted whereas 46% children were stunted at 18–23 months of age. Moreover, prevalence of stunting was higher among male (37%) compared to female children (35%) [17]. It is evident from global data that timing of stunting follows a universal trend. A comparison of childhood growth patterns in 54 LMICs reported that foremost growth faltering happened from 3 to 18–24 months of age [11]. From a Bangladeshi study, Hong et al. [82] reported that, 43% children aged 0–59 months were stunted with significantly less prevalence during the first 12 months of life but increased sharply during 12–23 months of age, which echoes with our finding.

There is a notion that male children are more valued in certain communities which is also related to their socio-economic condition. Therefore in such community, a male child is fed more nutritious food than a female child and presumed to have better nutritional status. However, we found that the male children are more susceptible to chronic undernutrition in our studied slum area. Several studies done in low socio-economic context reported similar results that male children are more prone to become stunted than female [4, 83, 84]. A meta-analysis from Africa found that boys are more stunted [85] than girls. Epidemiological studies in neonatology and in cohorts of pre-term infants and children, reported increased morbidity and mortality rate among males in early life [86–88]. Also, biologically programmed growth trajectory for male infants is greater than that for females [89] which consequently causes higher demand for most of the nutrients. Therefore, if this increased demand is not met adequately, male children will suffer from deficiency sooner and more frequently compared to female which may partly explain the relationship between sex and stunting.

### Limitation of the study

The key limitation of our study is sample size. Even though we enrolled 265 children at birth, loss to follow up of few children was unavoidable considering highly mobile slum population. By the age of 24 months, we had information from 211 children only. Considering the sample size, this study might have missed to identify the effect of dietary macronutrient intake on stunting. Additionally generalizability of the study findings may be another limitation. Population living in slums is significantly diverse and varied both economically and in terms of living conditions which result in deleterious health outcomes. Slum children in Bangladesh is the most disadvantaged group in terms of wellbeing compared to rural and non-slum urban areas. Therefore result of our study should be interpreted taking into account such vulnerable context.

## Conclusion

Stunting is the consequence of synergistic effect of diverse factors. This study aimed to estimate the usual macronutrient intake from complementary feeding and explore the factors related to stunting among a cohort of < 2 years aged children residing in a vulnerable slum area. Although average calorie intake, animal source protein and fat consumption were inadequate suggesting poor quality of complementary feeding practice in the studied population, diet was not a significant predictor of stunting between 12 and 24 months of age. Rather, standardized birth length along with male gender and poor socio-economic condition were the prominent determinants of stunting among these slum dwelling children. The mechanism of stunting begins even before a child is born and preventive measurement should be initiated focusing on life course approach. Moreover to effectively tackle chronic undernutrition, multipronged approach would be needed involving both the social development and nutrition-based interventions. Government and policymakers have to develop sustainable strategies to improve various social and environmental factors those are closely interrelated with chronic undernutrition particularly concentrating on urban slum areas. These are the people who are suffering the most and this is the population that will bear substantial burden of stunting in coming future if not properly intervened. Therefore priorities must be given to the deprived mothers and children living in the slum areas if we really want to reduce the prevalence of stunting in the country.

## Abbreviations

ABF: Average breastfeeding frequency/day
ADD: Average days suffered from diarrhoea/month
AOR: Adjusted odds ratios
BDHS: Bangladesh demographic and health survey
CI: Confidence interval
DDS: Dietary diversity score
EBF: Exclusive breastfeeding
GEE: Generalized estimating equation
Icddr,b: International Center for Diarrhoeal Disease Research, Bangladesh
IQR: Interquartile range
LAZ: Length for age Z score
LMICs: Low and middle income countries
MAL-ED: The Interactions of Malnutrition & Enteric Infections: Consequences for Child Health and Development
MDD: Minimum dietary diversity
MDGs: Millennium development goals
MICS: Multiple Indicator Cluster Survey
ORS: Oral rehydration solution
SES: Socioeconomic status
UNICEF: United Nations Children’s Fund
VIF: Variance inflation factor
WAZ: Weight for age Z score
WHO: World Health Organization
